# Role of kinesins in directed adenovirus transport and cytoplasmic exploration

**DOI:** 10.1371/journal.ppat.1007055

**Published:** 2018-05-21

**Authors:** Jie Zhou, Julian Scherer, Julie Yi, Richard B. Vallee

**Affiliations:** 1 Department of Biological Sciences, Columbia University, New York City, New York, United States of America; 2 Department of Pathology and Cell Biology, Columbia University, New York City, New York, United States of America; Tufts University School of Medicine, UNITED STATES

## Abstract

Many viruses, including adenovirus, exhibit bidirectional transport along microtubules following cell entry. Cytoplasmic dynein is responsible for microtubule minus end transport of adenovirus capsids after endosomal escape. However, the identity and roles of the opposing plus end-directed motor(s) remain unknown. We performed an RNAi screen of 38 kinesins, which implicated Kif5B (kinesin-1 family) and additional minor kinesins in adenovirus 5 (Ad5) capsid translocation. Kif5B RNAi markedly increased centrosome accumulation of incoming Ad5 capsids in human A549 pulmonary epithelial cells within the first 30 min post infection, an effect dramatically enhanced by blocking Ad5 nuclear pore targeting using leptomycin B. The Kif5B RNAi phenotype was rescued by expression of RNAi-resistant Kif5A, B, or C, and Kif4A. Kif5B RNAi also inhibited a novel form of microtubule-based “assisted-diffusion” behavior which was apparent between 30 and 60 min p.i. We found the major capsid protein penton base (PB) to recruit kinesin-1, distinct from the hexon role we previously identified for cytoplasmic dynein binding. We propose that adenovirus uses independently recruited kinesin and dynein for directed transport and for a more random microtubule-based assisted diffusion behavior to fully explore the cytoplasm before docking at the nucleus, a mechanism of potential importance for physiological cargoes as well.

## Author summary

The role of plus-end directed microtubule motors in virus entry into host cells is a long-standing question. In this study, the authors identify the kinesins responsible for adenovirus plus end-directed transport along microtubules, the mechanism for kinesin recruitment, and both directed and motor-based exploratory movements involved in adenovirus’ search for the nucleus.

## Introduction

Viruses generally depend on active transport inside host cells as the crowded cytoplasm restricts their ability to diffuse [1, 2]. Viruses have evolved mechanisms to hijack microtubule motor proteins for this purpose during cell entry and egress [3]. Adenovirus [4, 5], herpesvirus [6], vaccinia [7], adeno-associated virus [8] and HIV-1 [9, 10] each exhibit bidirectional movements along microtubules (MTs), consistent with possible use of both minus- and plus- directed microtubule motors. Cytoplasmic dynein, in particular, has been implicated in microtubule minus end-directed transport for several viruses, but less is known about the contributions of kinesins [11].

The human adenoviruses are non-enveloped, dsDNA-containing particles, consisting of more than 57 serotypes grouped into seven species [12]. Adenovirus infections are usually self-limiting, but can have fatal outcomes in immunocompromised patients. However, engineered versions are preferred vehicles for vaccine delivery and therapeutic gene transfer [13]. Adenovirus enters target cells by receptor-mediated endocytosis [14]. Following endosomal escape, Ad particles travel along microtubules (MT), and then dock at nuclear pore complexes (NPC) to deliver their DNA genome into the nucleus [4, 15, 16]. In enucleated cells or those treated with the nuclear export inhibitor leptomycin B, capsids bypass the nucleus and accumulate in the vicinity of the centrosome [17, 18]. Transport to the cell center involves the MT minus end-directed motor protein cytoplasmic dynein, and can be inhibited by microtubule-destabilizing agents or blocking dynein or dynactin function using dominant negative cDNAs, RNAi, or acutely injected function-blocking antibodies and inhibitory fragments directed at dynein subunits [4, 5, 15, 19, 20].

We have found that cytoplasmic dynein is directly recruited to adenovirus by its major capsid subunit hexon, via the dynein intermediate and light intermediate chains (ICs; LICs) and without the need for adaptor proteins used by physiological forms of dynein cargo [5]. We found in addition that exposure of hexon to reduced pH, as would happen during passage through early endosomes, triggers a reversible change in the hexon hypervariable region 1 (HVR1), to activate dynein recruitment [21]. This function is also markedly stimulated by PKA, which phosphorylates LIC1 and enhances dynein binding to the virus, while releasing the motor protein from endogenous cargo sites associated with lysosomes [19].

The role of kinesins in adenovirus transport is less well understood. A form of kinesin-1, Kif5C, has been reported to function in disruption of Ad5 capsids and associated nuclear pore complexes to facilitate genome transfer into the nucleus [22]. However, kinesins responsible for adenovirus plus-end directed transport along MTs were not evaluated and remain to be identified.

In the current study, we performed an RNAi screen of all 38 plus-end directed transport kinesins in the human genome. We identified Kif5B as the major motor responsible for plus end-directed adenovirus transport, along with several minor kinesins which may contribute to this role. Kif5B interacted with the capsid subunit penton base (PB), independent of hexon or low pH exposure. Kif5B RNAi caused a marked increase in microtubule minus end-directed transport toward the cell center, and a resulting accumulation of Ad5 particles in the pericentrosomal region. Following this period in normal or kinesin-knockdown cells, adenovirus transport along microtubules was more randomly directed, though the magnitude of this cytoplasmic exploration behavior was also reduced by Kif5B knockdown. These results suggest distinct evolution of dynein and kinesin recruitment to adenovirus and a coordinated requirement for the two motors during virus entry.

## Results

### Screen for kinesins in adenovirus transport

**Fig 1:**
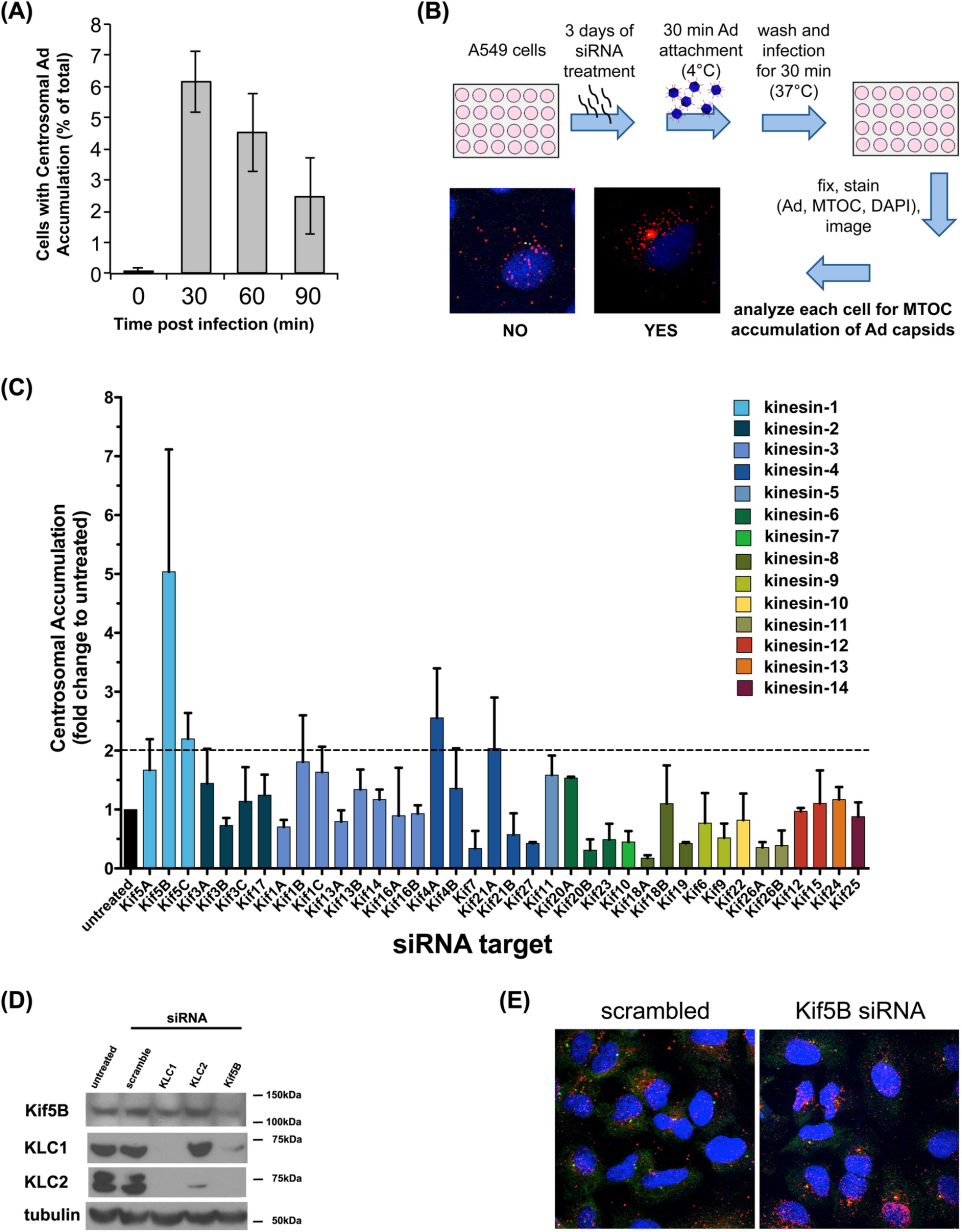
Screen for kinesins responsible for Ad5 transport during early infection. (A) Quantification of Ad5 distribution in control A549 cells at indicated times post-infection (p.i.). Error bars represent standard deviation from three independent experiments. Pericentrosomal accumulation peaks at ~30min p.i., whereas nuclear accumulation reaches its maximum at ~90min p.i. (B) Schematic representation of the RNAi screening procedure with control and Kif5B fluorescence microscopic examples of Ad5-infected A549 cells exhibiting dispersed vs. pericentrosomally concentrated at 30 min p.i. (C) Analysis of pericentrosomal Ad5 capsid accumulation at 30min p.i. after siRNA treatment for indicated 38 kinesin heavy chain genes; displayed as fold change compared to scrambled siRNA condition. Error bars represent standard deviation from three independent sets of screening results. Dotted line indicates two-fold increase in pericentrosomal accumulation over scrambled control. (D) Western blot of A549 cells untreated or treated with scrambled, KLC1, KLC2, or Kif5B siRNA for 3 days. Knockdown efficiency was tested by immunoblotting with anti-KLC1, -KLC2, and -Kif5B antibodies. Blotting with anti-tubulin antibody as loading control. We note that KLC1 siRNA also depleted KLC2. (E) Representative micrographs showing Ad5 distribution in A549 cells, which were pre-treated with scrambled or Kif5B siRNAs and infected with purified Ad5. Cells were fixed at 30min p.i. and subsequently stained with anti-hexon (Ad5 capsid), anti-γ-tubulin (centrosome) antibodies, and counter-stained with DAPI.

As a first step toward identifying microtubule plus end-directed kinesins for Ad5 transport, we quantified virus redistribution to the nucleus and the pericentrosomal region in human lung epithelial A549 cells at 0, 30, 60 and 90 min post infection (p.i.). Ad5 particles could be seen to accumulate in the pericentrosomal region of a small subset of cells by 30min p.i. (Fig 1A), well before maximal targeting to the nuclear envelope, which usually occurs by 60 min [5].

We reasoned that depletion of an Ad5 transport kinesin might, in principle, lead to an increase in dynein-driven Ad5 accumulation in the pericentrosomal region. We tested this possibility with an siRNA library targeting the heavy chains of each of the known 38 plus end-directed human kinesins, covering all 14 families. Three days following siRNA treatment, we infected the cells with Ad5 and determined the percentage of cells showing pericentrosomal Ad5 capsid accumulation at 30 min p.i. (Fig 1B and 1C).

Reduction of the level of Kif5B, a well-known member of the kinesin-1 subfamily, had the most pronounced effect on virus redistribution, with a 5-fold increase in cells exhibiting clear pericentrosomal virus accumulation (Fig 1C). The level of Kif5B was reduced by ~70% (Fig 1D), as tested with an isoform specific antibody (S1A Fig). Incomplete reduction, however, may still be responsible for some of the residual pericentrosomal targeting we observed (Fig 1E). RNAi directed against the two other kinesin-1 heavy chain isoforms, Kif5A and Kif5C, also showed significant, though weaker, effects on virus redistribution to this region of the cell (Fig 1C). In addition, we observed increased pericentrosomal accumulation with knockdown of kinesin-4 subfamily heavy chain genes, Kif4A and Kif21A.

### Role of kinesins in pericentrosomal-specific Ad5 targeting

**Fig 2:**
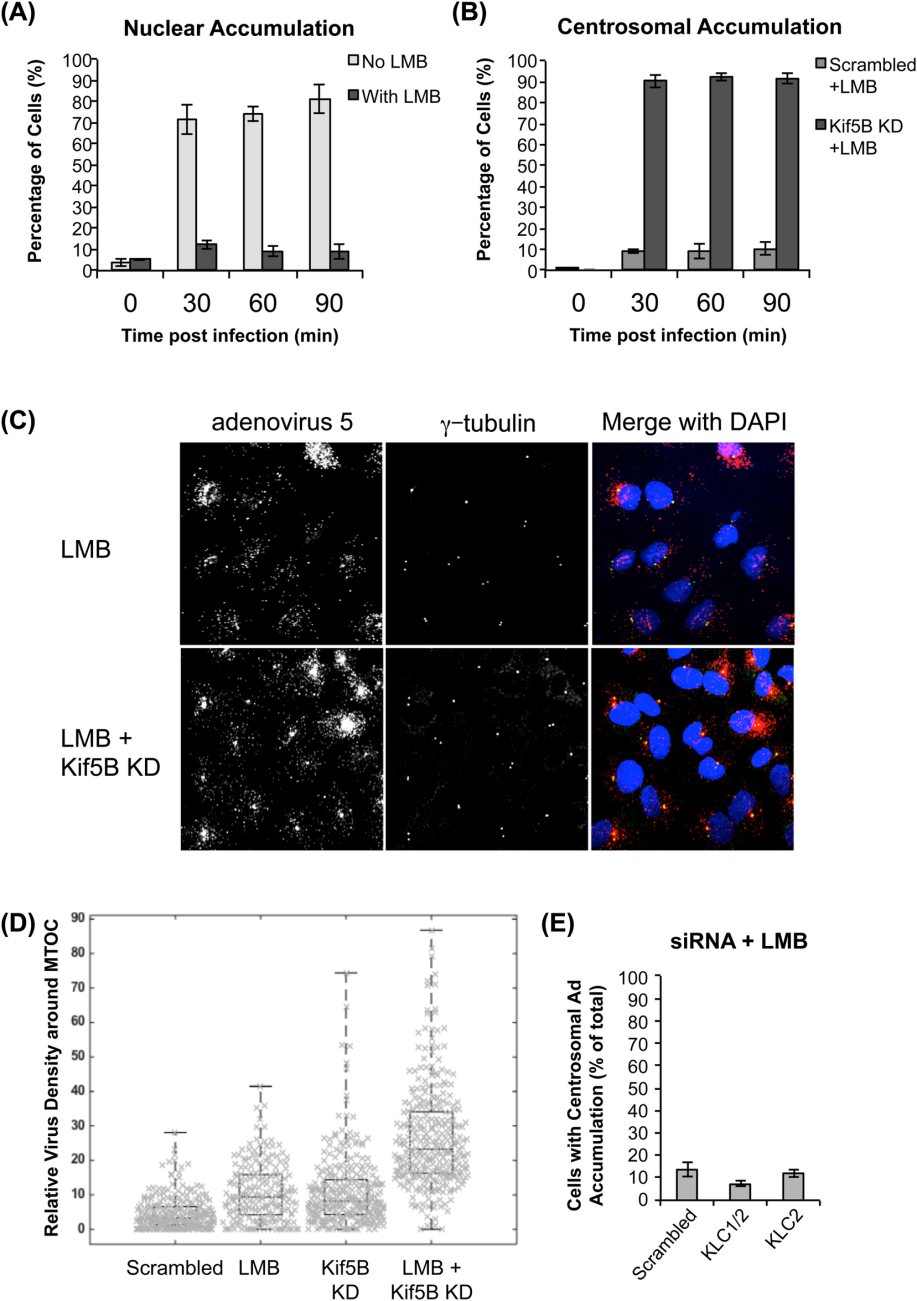
Effect of leptomycin B on Ad5 distribution in the presence of kinesin subunit RNAi. (A) Quantification of Ad5 nuclear distribution in A549 cells treated with or without leptomycin B (LMB) at indicated times post infection; bars represent standard deviation (N = 3). LMB treatment abolishes nuclear accumulation. (B) Quantification of Ad5 centrosomal distribution in LMB treated cells with Kif5B or scramble control siRNA treatment at indicated times post infection; bars represent standard deviation (N = 3). Kif5B knockdown in LMB-treated cells increases pericentrosomal accumulation 10-fold compared to scramble control. (C) Representative micrographs showing Ad5 distribution in A549 cells, which were LMB-treated with or without Kif5B siRNAs and infected with purified Ad5. Cells were fixed at 30min p.i. and subsequently stained with anti-hexon, anti-γ-tubulin antibodies, and counter-stained with DAPI. (D) Beeswarm plot of relative pericentrosomal virus density (see Materials and Methods) from multiple Ad5-infected cells pre-exposed to scrambled and Kif5B siRNAs, with our without LMB exposure. The top, middle, and bottoms lines in each box are the 25th, average, and 75th percentile, respectively. Combined treatment of cells with LMB and Kif5B siRNA shows a synergistic effect on increased relative pericentrosomal virus density. (E) Quantification of Ad5 distribution in A549 treated with LMB and KLC1 or KLC2 siRNA cells at 30min p.i.; bars represent standard deviation from three independent experiments.

Although the nucleus is the physiological destination for in-coming capsids [23], we reasoned that the attachment of the virus to the nucleus terminates motility and complicates analysis of underlying transport mechanisms. We, therefore, treated cells with the nuclear export inhibitor leptomycin B (LMB), which blocks adenovirus binding to nucleoporins [18]. Upon LMB treatment, the percentage of cells exhibiting nuclear accumulation markedly decreased (Fig 2A). However, the number of cells exhibiting pericentrosomal virus accumulation at 30 min p.i. increased modestly (from 6.1% to 9.1%) (Figs 1A and 2B). Importantly, the combination of LMB treatment with Kif5B knockdown led to a ~10-fold increased fraction of cells showing pericentrosomal virus accumulation (9.1% to 90.1%) (Fig 2B and 2C). We also quantified the concentration of individual virus particles reaching the MT organizing center (MTOC) by measuring the capsid density in the pericentrosomal region (see Materials and Methods). Although there was considerable variation between cells, this analysis again revealed strong enhancement of Ad5 redistribution to the pericentrosomal region upon Kif5B RNAi in LMB treated cells (Fig 2D). These results imply that Kif5B contributes substantially to Ad5 transport, normally serving in virus transport away from the cell center.

Physiological cargo recognition by kinesin-1 is often mediated through its light chains (KLCs). Indeed, KLCs were previously reported to interact with Ad protein IX (pIX), as part of the mechanism mediating nuclear pore binding and disruption [22]. We noted that Kif5B knock down also reduced KLC1 and KLC2 protein levels (Fig 1D), suggesting the HC-LC interaction stabilizes the LCs. However, to test more directly for an LC contribution to Ad5 behavior, we also performed RNAi for the individual KLCs. Knockdown of KLC1 also depleted KLC2, despite the sequence specificity of the siRNAs used, whereas KLC2 siRNA was specific for this isoform alone (Fig 1D). Knockdown in neither case had any detectable effect on pericentrosomal virus accumulation in the presence of LMB (Figs 2E and S1B). Thus, our results support a direct role for the heavy chain in kinesin-1 recruitment to the virus capsid and subsequent transport, whereas KLC1 and KLC2 are dispensable for these functions.

**Fig 3:**
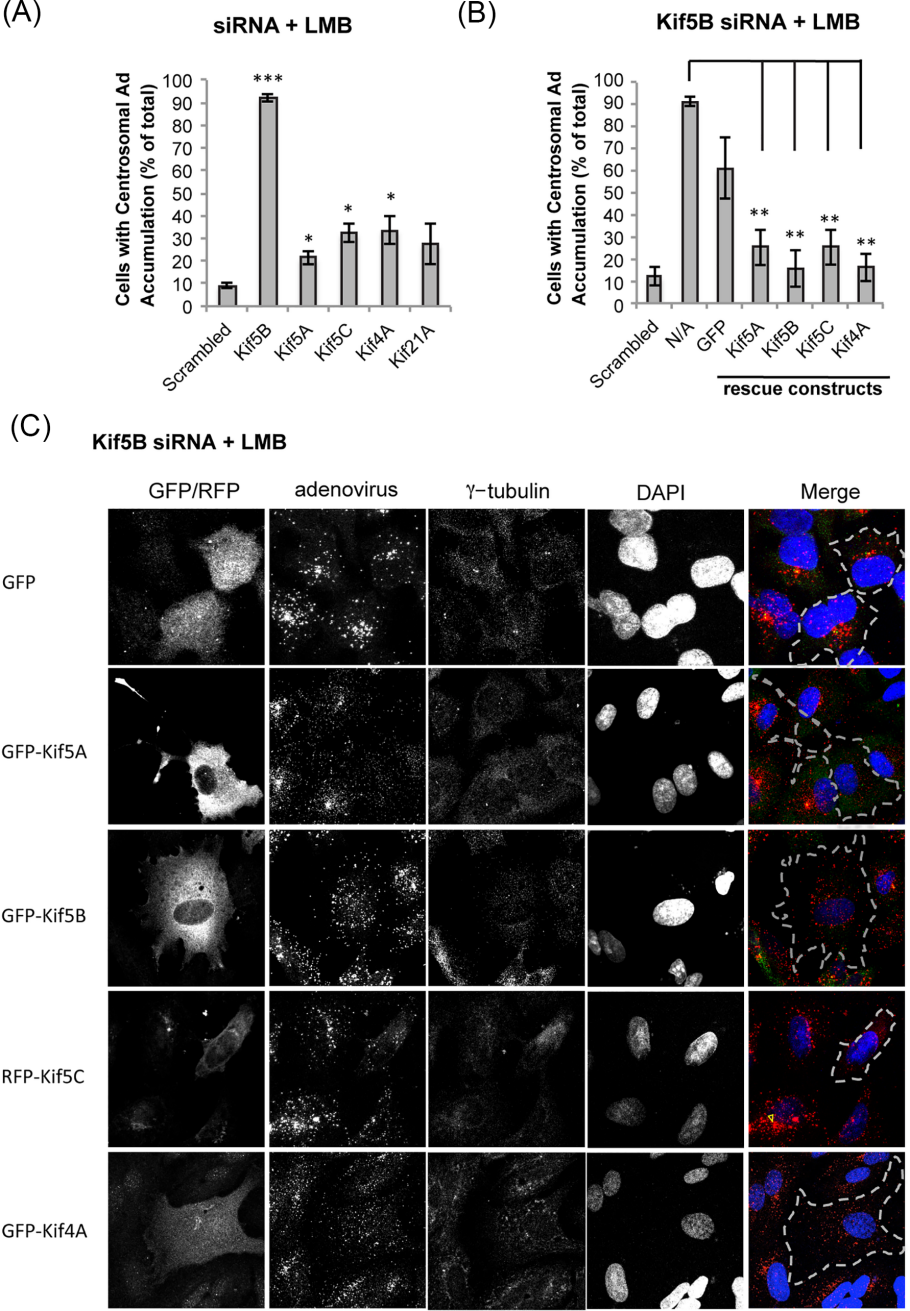
Effect of combined treatment with leptomycin B, kinesin RNAi, and Kif5 rescue construct expression on Ad5 distribution. (A) Quantification of Ad5 pericentrosomal accumulation in A549 cells treated with leptomycin B (LMB) and siRNAs for kinesin heavy chains implicated in Ad5 transport at 30min p.i.; bars represent standard deviation (N = 3), Different stars indicate significant differences in kinesin siRNA treatment means from scrambled siRNA treatment means. (*: P < 0.05, **: P<0.01; ***: P<0.001). Kif5A, Kif5B, Kif5C, Kif4A, and Kif21A RNAi all lead to increases in pericentrosomal Ad5 accumulation with Kif5B RNAi showing the strongest effect. (B) For Kif5B RNAi rescue analysis, A549 cells were treated with Kif5B RNAi and LMB as above, but also transfected with cDNAs encoding RNAi-insensitive kinesin isoforms. Quantification as in (A). (C) Representative micrographs showing Ad5 distribution in A549 cells, which were treated with LMB and Kif5B siRNA, and transfected with indicated cDNAs before infection with purified Ad5. Cells were fixed at 30min p.i. and subsequently stained with anti-hexon, anti-γ-tubulin, anti-GFP or -RFP antibodies, and counter-stained with DAPI.

We also tested the combined effect of LMB treatment with RNAi directed at additional kinesin heavy chain candidates. RNAi against Kif5A, Kif5C and Kif4A each increased the number of infected cells showing pericentrosomal Ad5 accumulation by 2-3-fold in the presence of LMB compared to a scrambled RNAi control (Figs 3A and S1C). We note that Kif5A and C are expressed at much lower levels than Kif5B in A549 cells (Source: Protein Abundance Database, http://pax-db.org/), suggesting that all three isoforms might contribute to Ad5 transport, but, perhaps, in proportion to their abundance. To test the functional relationship between the isoforms and as a control for potential off-target effects of Kif5B RNAi, we determined the ability of each of the Kif5 isoforms to compensate for Kif5B knockdown. We observed clear rescue in each case, suggesting that the three kinesin-1 isoforms contribute to a common function, though with the most abundant form, Kif5B, playing by far the most substantial role in Ad5 transport (Figs 3B, 3C and S1D). Interestingly, Kif4A overexpression partially rescued the effects of Kif5B knockdown on virus redistribution (Fig 3B and 3C). Kif21A rescue was not tested in light of its relatively minor contribution.

### Contribution of Kif5B to adenovirus behavior in live cells

**Fig 4:**
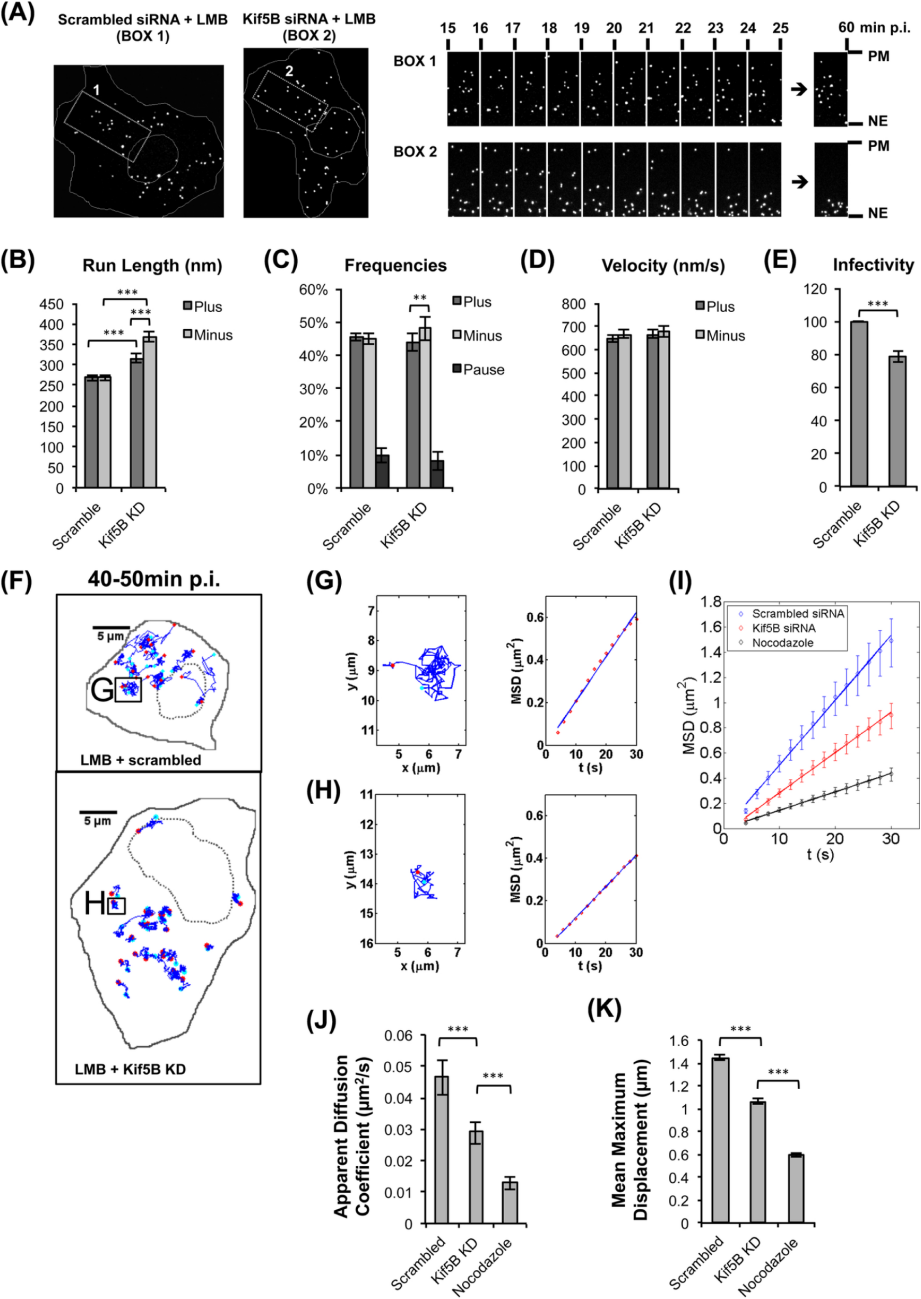
Effect of Kif5B RNAi on assisted diffusion during Ad5 transport. (A) Time series of LMB treated A549 cells infected with Alexa-546-Ad5. To test for redistribution to the pericentrosomal region, virus particles in the boxed areas in scrambled control and Kif5B siRNA treated cells were monitored live for 60 min at 30 second intervals (see also S1 and S2 Movies). Boxed regions span from the nuclear envelope (NE) to the plasma membrane (PM), and indicated accelerated accumulation of virus particles towards the NE in Kif5B knockdown cells. (B-D) High-resolution live imaging (23frames/sec) for 2min 10-30min p.i. and automated particle tracking analysis [5] of Alexa-546-Ad5 infected A549 cells treated with scramble control or Kif5B siRNA reveals (B) run length, (C) frequency, and (D) velocity of Ad5 movements. Error bars show 95% confidence intervals. *: P < 0.05, **: P<0.01; ***: P<0.001. Whereas velocities remain unaffected by Kif5B RNAi, MT minus end-directed run frequencies, as well as minus and plus end-directed run lengths increase. (E) Quantification of Ad5 infectivity in control and Kif5B knockdown 293A cells revealed by fluorescent focus assay; bars represent standard deviation (N = 3). ***: P<0.001. Kif5B siRNA leads to a ~20% decrease in Ad5 infectivity. (F) Examples of Ad5 tracks from control and Kif5B knockdown cells at 40-50min p.i. (recorded at 0.5 frames/sec). Individual viruses exhibited a stochastic wandering behavior in both control and Kif5B knock down cells, but the extent of exploration was reduced by Kif5B RNAi. The starting and end points of each track are labeled in cyan and red, respectively. (G-H) Left panels show enlarged representations of the boxed Ad5 track in (G) control and (H) Kif5B knock down cells. Right panels show mean square displacement (MSD) for each track. (I) MSD values as a function of time >300 tracks in nocodazole treated and scrambled or Kif5B siRNA treated cells, with 95% confidence interval indicated with error bars. A reduced slope for Kif5B knockdown is consistent with assisted diffusion. (J-K) Average of values for apparent diffusion coefficient (J) and maximal displacement (K) of Ad5 tracks analyzed in (I). Error bars indicate 95% confidence intervals. *: P < 0.05, **: P<0.01; ***: P<0.001.

To test the role of Kif5B in adenovirus behavior more directly, we performed continuous imaging of Alexa-546-labeled Ad5 in live, LMB-treated A549 cells from 15 to 60 min p.i. at 30 second intervals. Net particle movement towards the pericentrosomal region was observed in 13 out of 23 Kif5B siRNA-treated cells, compared to 2 out of 24 scrambled siRNA- treated cells (Fig 4A, S1 and S2 Movies). The net inward movement was most prominent from 15–30 min p.i. (Fig 4A and S2 Movie), supporting a normal role for Kif5B in MT plus end-directed Ad5 transport during the early stages of infection.

We then examined individual virus capsid motility at higher temporal resolution (23 frames/second, fps) over 2 min intervals from 15–30 min p.i., a period when capsids undergo the greatest redistribution toward the centrosome in LMB-treated cells. Tracings for stationary particles or those exhibiting small-scale Brownian motion are filtered out as part of the particle-tracking analysis [5]. In Kif5B knockdown cells, we observed a pronounced increase in inward minus-end directed run length (Fig 4B). Small, though statistically significant, increases in outward, MT plus-end directed run length and in the relative frequency of plus- *vs*. minus-end runs and pauses could also, however, be detected (Fig 4C). Virus transport velocity was unaffected (Fig 4D). The persistence of outward runs in Kif5B knockdown cells (Fig 4B, 4C and 4D) despite the global effects of capsid translocation towards the centrosomal region, is consistent with incomplete Kif5b knockdown (Fig 1D). A minimal change in run length has previously been observed from kinesin inhibition in *Drosophila* embryonic lipid droplet motility (Shubeita et al., 2008), and may reflect compensatory effects on dynein behavior and the small number of motor protein molecules involved in transport of individual cargo (see Discussion). Using a fluorescence focus assay [24], we also observed an ~20% inhibitory effect of Kif5b knockdown on Ad5 infectivity (Fig 4E). This, too, is a relatively small effect, but consistent with the limited impact of microtubule motor-driven transport on measures of whole cell physiological changes (see below).

We also monitored the effects of Kif5B RNAi on virus behavior later in infection (30–60 min), when directed movements are still seen, but the distribution of virus in the LMB-treated cells has reached a steady state, with no clear bias in transport toward or away from the cell center. To analyze this behavior we employed a different imaging regimen, examining Ad5 capsids at 0.5 fps for 10min between 30 and 60 min p. i. (Fig 4F). At this time scale, we observed numerous capsids exhibiting non-directional “wandering” behavior within the cytoplasm (Fig 4F). Local virus movements appeared to be linear, but random over-all with regard to direction and distance. This was confirmed by analysis of mean squared displacement (MSD), which we found to increase linearly with time (Fig 4G and 4H). This behavior is characteristic of random motility, such as Brownian movement, but the scale of over-all virus transport was greater than expected for simple diffusion. Knockdown of Kif5B resulted in a clear decrease in MSD values (Fig 4I and 4J), indicating that capsids explore a smaller region of the cell under this condition. To test the dependence of the stochastic virus movements on MTs further we exposed cells to the MT depolymerizing drug nocodazole, which dramatically reduced virus wandering behavior. The apparent diffusion coefficient for the virus particles obtained from the slope of MSD plot was more than 4-fold lower than in control cells (Fig 4I and 4J), and the maximum displacement (distance between starting and ending positions) was halved. Thus, together, our data support at least two active MT-based modes of for Ad5 capsid movement: directed transport and assisted diffusion.

### Physical association of adenovirus with Kif5B

**Fig 5:**
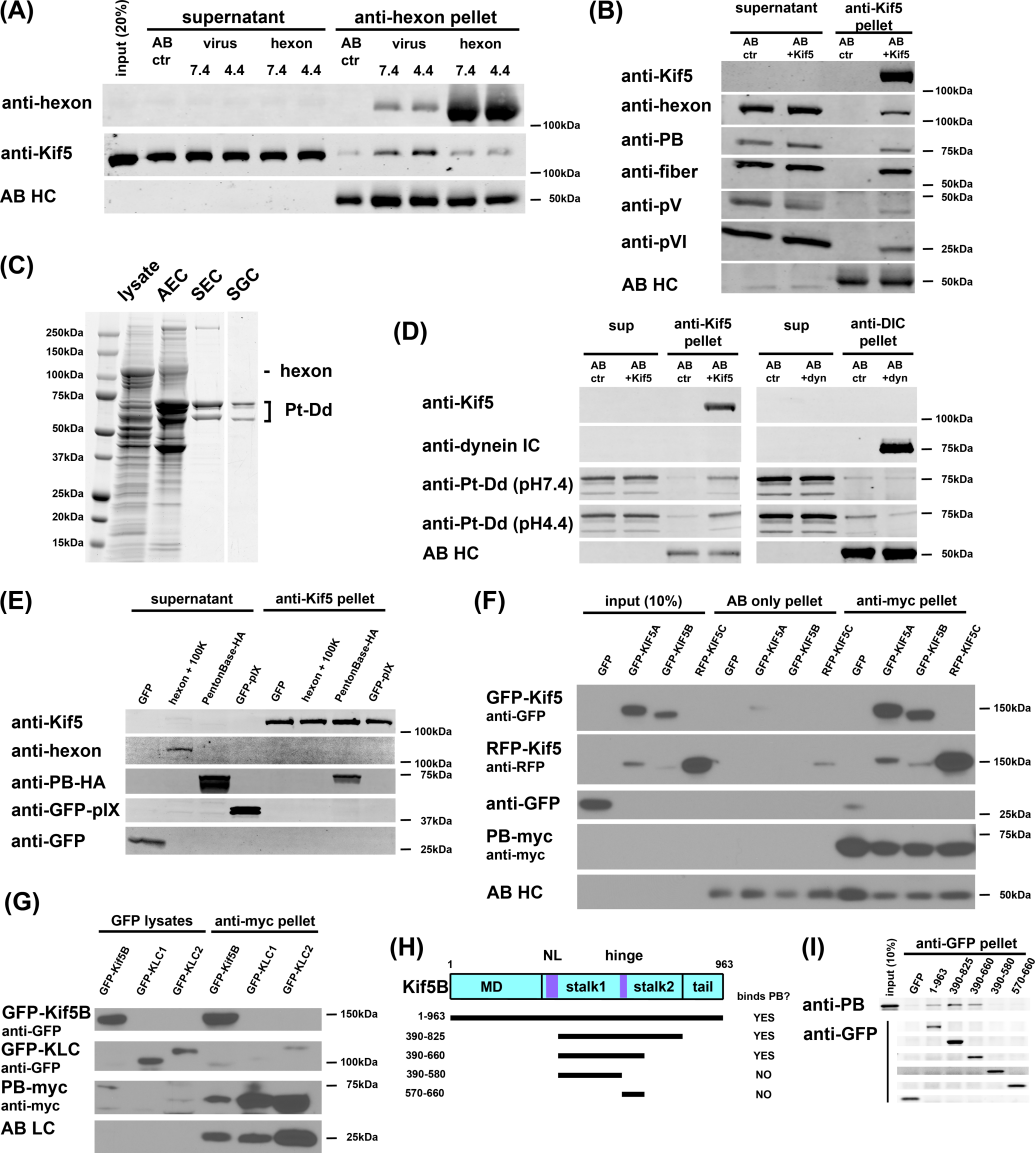
Interaction between adenovirus and kinesin-1. (A) Immunoisolated Ad5 capsids or hexon alone were transiently exposed to pH7.4 or 4.4 buffer, and then incubated at pH 7.4 with a rat brain kinesin fraction. Kinesin-1 heavy chain (Kif5) coimmunoprecitated with adenovirus capsids, but not with hexon alone, and independent of pH. AB HC: antibody heavy chain. (B) Coimmunoprecipitation of Ad5 capsids with kinesin-1, isolated from brain kinesin fraction using H2 monoclonal antibody. Capsid components in immunoprecipitate included penton base, PB: protein V, pVI: protein VI. (C) Purification of Ad5 penton-dodecahedra (Pt-Dd) from infected 293A cell lysate, stained with Coomassie blue, show preparation following anion exchange chromatography (AEC), size exclusion chromatography (SEC), and sucrose gradient centrifugation (SGC). (D) Immunoisolated kinesin-1 (MAB1614) but not cytoplasmic dynein (MAB1618) pulled-down purified Pt-Dd, independent of Pt-Dd exposure to low pH. (E) Immunoisolated kinesin-1 (MAB1614) pull-down recombinant penton base, but not hexon, pIX or GFP expressed in 293A cells for 24h. (F) Penton-base pull-down of kinesin. Myc-tagged penton base was coexpressed with GFP, GFP-Kif5A, GFP-Kif5B, and RFP-Kif5C in 293A cells for 24h and lysates were subjected to anti-myc immunoprecipitation. All three Kif5 isoforms coimmunoprecipitated with myc-penton base to similar levels. (G) GFP-tagged Kif5B heavy chain and the kinesin light chains KLC1 and KLC2 were coexpressed with penton base-myc in 293A cells for 24h and lysates were subjected to anti-GFP immunoprecipitation. Penton base coimmunoprecipitated with Kif5B, but neither kinesin light chain. (H) Schematic representation of the KHC (KIF5B) polypeptide and summary of the penton base binding result (data in panel I). Penton base bound Kif5B constructs, which contain the central hinge region between stalk 1 and stalk 2. MD: motor domain, NL: neck linker. (I) GFP-Kif5B truncation constructs were expressed in 293A cells and lysates were subjected to anti-GFP immunoprecipitation. Upon addition of purified Pt-Dd, binding was assessed by Western blotting with anti-GFP and anti-PB antibodies.

We previously found that cytoplasmic dynein is recruited to Ad5 by the major capsid subunit hexon, primed for binding by exposure to low pH in the endosome [5, 21]. To define the mechanism for Kif5B recruitment we tested for a physical interaction with capsid subunits. We used an anti-hexon monoclonal antibody to immuno-purify intact Ad5 capsids or hexon alone [21], with and without transient low pH exposure. We then tested for co-immunoprecipitation of kinesins from a GTP-release fraction of MT binding proteins isolated from rat brain cytosolic extracts [25]. Kinesin-1 could be readily seen to co-immunoprecipitate with Ad5 capsids, but not with hexon. In contrast to dynein [5, 21], the kinesin-1-virus interaction was unaffected by prior exposure of capsids to reduced pH (Fig 5A). In reciprocal experiments, we also found Ad5 capsids to co-immunoprecipitate with kinesin-1 (Fig 5B).

The absence of a hexon-kinesin-1 interaction suggested the involvement of a different capsid protein. Potential candidates should, in principle, be exposed at the capsid surface during cytoplasmic transport, and would include the major capsid subunit penton base and the minor capsid protein IX, which has been reported to interact with KLC1 [22]. Notably, the third major capsid component, fiber, is lost during initial endocytosis of the virus [26]. Absolute levels of penton base co-immunoprecipitating with adenovirus virions have been found to decline with time p.i. However, relative levels appeared less affected. In fact, this subunit was still readily detected at > 1 hr p.i. by this approach [27] and by immunocytochemistry of infected cells [5, 28, 29]. Based on these considerations we first tested a penton base-containing subassembly for kinesin-1 binding. We isolated “penton dodecahedron” complexes (Pt-Dd) found in Ad-infected cells following lysis, which are composed of an ordered assembly of penton base and fiber subunits [30] (Fig 5C) and organized in a manner closely resembling that in the complete Ad capsid [31]. We clearly detected Pt-Dd in kinesin-1, but not in cytoplasmic dynein, pull-downs (Fig 5D). Given the unlikely role for fiber in kinesin-1 recruitment, these results suggest that Ad5 interacts with kinesin-1 through penton base. We also expressed individual capsid subunits in 293A cells and found that kinesin-1 co-immunoprecipitated with HA-tagged penton base, but not hexon or pIX (Fig 5E). To test the ability of the three Kif5 isoforms to bind to penton base, we co-expressed myc-tagged penton base with fluorescent protein-tagged versions of Kif5A, B, or C in 293A cells. After anti-myc IPs of the cell lysates, we detected similar amounts of each of the three isoforms, but not GFP alone (Fig 5F), in the pellet fraction, consistent with the ability of each of the three kinesin-1 isoforms to transport adenovirus (Fig 3B and 3C). We also co-expressed myc-tagged penton base together with the kinesin light chains GFP-KLC1 and -KLC2. We saw no detectable interaction between penton base the kinesin light chains (Fig 5G), consistent with our KLC RNAi analysis (Fig 2E).

Each of the kinesin-1 heavy chain polypeptides consists of an N-terminal motor domain, followed by a neck-linker region, and highly elongated coiled-coil α-helical stalk and tail domains (Fig 5H). The stalk consists of two coiled-coil regions separated by a predicted hinge domain. The tail mediates physiological cargo interactions either directly or through the kinesin light chains [32–34]. To define the penton base binding site within the kinesin-1 heavy chain, we generated GFP-tagged fragments of the Kif5B heavy chain (Fig 5H). Only constructs spanning the hinge region showed a detectable interaction with expressed penton base (Fig 5I). These results indicate that the interaction is mediated by the central portion of the Kif5B stalk, possibly including the hinge region. We note that the penton base binding region partially overlaps with that for the physiological kinesin recruitment factor JIP1and may be involved in kinesin-1 auto-inhibition [35, 36].

## Discussion

**Fig 6:**
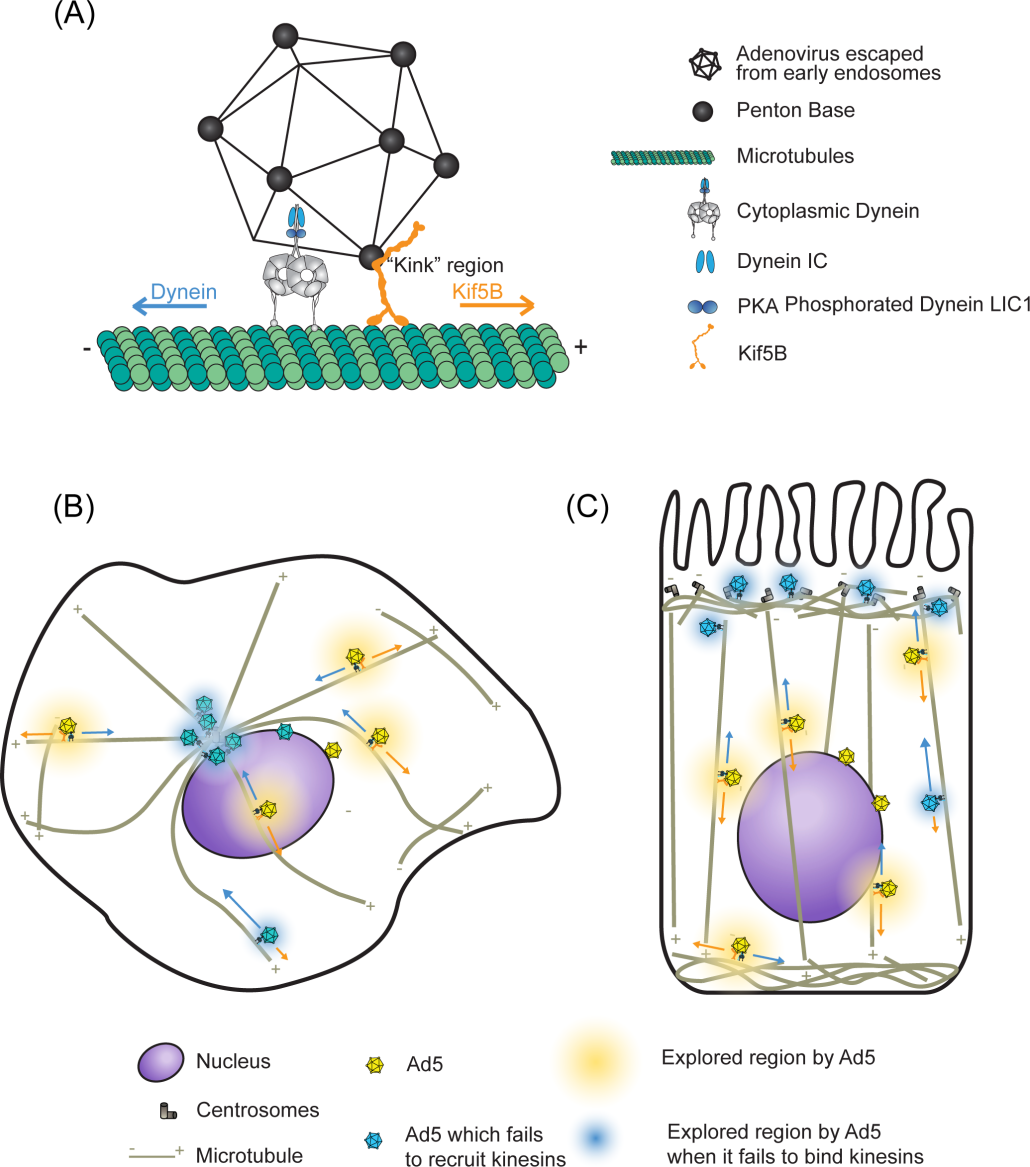
Cartoon representation of Ad5 –motor protein interactions and transport. (A) Adenovirus showing interactions with cytoplasmic dynein [5, 19] and kinesin-1 (this study). Although penton base levels may decline during infection [27], penton base remains clearly detectable on capsids throughout transport to the nucleus [5]. Adenovirus penton base is shown to recruit the kinesin heavy chain through the stalk region. (B) Adenovirus behavior in cultured A549 cell. Ad5 exhibits bidirectional transport mediated by cytoplasmic dynein and, based on the current study, kinesin-1, with minor assistance from other kinesins. Ad5 (yellow capsids) is able to actively explore a substantial region of the cytoplasm (yellow region) using a motor-driven assisted diffusion mechanism. Reduction of kinesin-1 protein levels by RNAi leads to Ad5 (cyan capsids) accumulation near the centrosome and less extensive exploration (blue region). (C) Presumptive adenovirus behavior in multi-ciliated airway epithelial cell. Bidirectional Ad5 transport may in this case be required for the virus to explore the apical-basal axis of the cell and more efficiently locate to the nucleus.

Adenovirus exhibits bidirectional transport along microtubules during the early stages of infection. Whereas MT minus end-directed Ad transport to the cell center is governed by cytoplasmic dynein, we have now identified a major role for the kinesin-1, Kif5B, in MT plus end-directed Ad transport, and minor contributions from additional kinesin isoforms. These results, together with our previous findings on cytoplasmic dynein [5, 19], provide the first detailed mechanism for bidirectional transport of incoming virus, with important implications for understanding the evolution of viral motor protein recruitment and the role of kinesins in infection (Fig 6A).

### Identification of Ad5 transport kinesins

A number of viruses have been found to rely on kinesin-mediated transport, but most studies have focused on a potential role for this class of motor proteins in virus egress [3, 37]. We have identified a role for Kif5B in Ad5 transport prior to capsid binding to the nucleus. For our analysis, we focused on the period between endosomal escape and nuclear envelope binding, when Ad5 transport is known to depend on motor proteins [16, 38, 39]. Using an RNAi screen of 38 MT plus end-directed kinesin heavy chains we have found Kif5B to be the predominant transport kinesin for Ad5. Our evidence also suggests a role for additional kinesins, including the other kinesin-1 family members Kif5A and Kif5C. Importantly, expression of each of the kinesin-1 isoforms rescued the effects of Kif5B RNAi, suggesting that Kif5A and C are fully capable of adenovirus transport, though they are expressed at too low concentrations to make an important quantitative contribution to this behavior.

The kinesin heavy chain Kif5C together with KLC1 and 2 has been implicated in another study in Ad2 capsid disassembly at the nuclear pore complex, involving a KLC1/2 interaction with the capsid subunit protein IX [22]. We find that knockdown of KLC1 and/or KLC2 had no detectable effect on the redistribution and transport of Ad5 capsids (Fig 2E), and see no evidence for an involvement of protein IX (Fig 5E). However, we find the major capsid protein penton base to mediate direct recruitment of the heavy chain of all three kinesin-1 isoforms (Fig 5F–5I). Based on findings by us and others [5, 27–29], penton base remains associated with Ad capsids throughout the cytoplasmic phase of capsid entry, and therefore, represents a plausible kinesin binding partner.

These data suggest that we have identified a novel kinesin role in capsid transport distinct from that for nuclear entry. Two kinesin-4 family members, Kif4A and Kif21A, were also implicated by our analysis in plus-end directed adenovirus transport. Both Kif4A and Kif21A have previously been reported to regulate microtubule dynamics and stabilization [40, 41]. Interestingly, Kif4A has been suggested to contribute to transport of HIV Gap proteins [42], and Kif4-mediated microtubule stabilization enhanced early infection of HIV-1 [43]. Whether these kinesins affect in-coming Ad5 redistribution (Fig 3A) through changes in MT stability or direct effects on virus transport is uncertain.

Of note, Ad replicates and assembles within the nucleus and induces lytic cell death to release and spread its progeny virions [44]. Thus, there is no apparent late function for kinesins in this system. The role we identify for Kif5B and the other kinesins found in our screen in early infection seems to be the predominant one in this system.

### Distinct mechanisms for kinesin-1 *vs*. dynein recruitment to adenovirus

Previously we reported that low pH-primed Ad5 directly recruits the cytoplasmic dynein IC and LIC1 subunits through the hexon hypervariable region HVR1 [19, 21]. Here, we identify a distinct kinesin-1 recruitment mechanism, which is independent of pH and hexon, but, instead, involves another major Ad5 capsid protein, penton base. We found this subunit to interact with the Kif5A, B, and C heavy chains, and mapped the penton base-binding site to the kinesin stalk. Although this region is not well conserved among the Kif5 isoforms, we suspect there must be sufficient structural conservation to support the penton base interaction in each case. Several cellular proteins have been found to interact directly with the kinesin heavy chain (see S1 Table), and not through the kinesin light chains [32]. Whereas most kinesin-1 heavy chain interactions occur through its tail domain, JIP1 can also bind within the stalk. Kinesin-1 can fold *via* its central hinge to allow the tail to bind to and inhibit the motor domain [35]. The region of the kinesin-1 stalk implicated by our analysis in penton base binding raises the possibility that Ad5 may have evolved to select for, or induce, the unfolded, activated kinesin-1 state.

### Role of Kif5B in Ad5 transport

From extensive live imaging we have noted that only about 10% of Ad5 capsids are motile within any given 5~10 second time window, though almost all virus particles show directed motility within a 10 min window, suggesting that kinesin recruitment or activity may be intermittent and/or limited. This possibility is supported by the short plus end-directed run lengths for adenovirus we observe *in vivo* similar to published run-lengths for purified individual kinesin-1 molecules (~1 um). Thus, we suspect that most viruses are transported by no more than a single kinesin, in contrast to physiological cargoes. We were unable to detect colocalization of endogenous or expressed kinesin with individual adenovirus particles by immunocytochemistry, perhaps consistent with the few motile particles at a given instant, and hampered by the high levels of free kinesin-1 in the cytoplasm. We do not observe the juxta-nuclear accumulations of kinesin-positive capsid structures previously reported [22].

Under conditions of reduced Kif5B expression we observed a significant increase in inward virus run length, though, curiously, a modest increase in outward run length as well. The latter observation is consistent with a previous report on the behavior of *Drosophila* embryo lipid droplets, which displayed slight, but statistically significant increases in plus-end run length, though a more significant increase in minus-end run length after kinesin-1 inhibition [45]. These results may reflect roles for opposite-directed motor proteins in run termination and in “tug-of-war-mediated motor activation” [46].

We proposed two temporally distinct transport roles for Kif5B during entry. Up to ~30 min p.i., Kif5B-mediated transport delays virus redistribution to the nucleus and retards infection. After ~30 min p.i., Kif5B-dependent assisted diffusion enables more efficient cytoplasmic exploration by the virus and stimulates infection. The first role is supported by a dramatic centrifugal redistribution effect of Kif5B knock down (Fig 2) by 30 min p.i., using LMB to block nuclear pore binding. The second role is supported by the virus motility analysis between 30–60 min p.i. (Fig 4F**–**4K).

### Evolutionary role of kinesin-1 in adenovirus infection

Adenovirus recruitment of cytoplasmic dynein would seem to provide the evolutionary advantage of aiding virus transport toward the nucleus, and this possibility is supported by a reduction in infectivity in dynein-inhibited cells [19]. We find that Kif5B knock-down also inhibits infectivity, though modestly. The basis for this outcome and its implications for the evolution of kinesin recruitment by Ad5 are uncertain.

One hypothesis is that kinesin-mediated virus transport evolved as a means to defend the cell from viral attack, keeping virus particles from reaching the cell center. This seems an unlikely model, as viruses defective in kinesin binding would have an evolutionary advantage, and should quickly have out-competed a kinesin-binding strain.

A second hypothesis is that kinesins are needed for full dynein activity. This possibility has been raised for peroxisome transport in Drosophila S2 cells [46] and the inward transport of HIV-1 [10]. However, we find that Kif5B knockdown actually *increases* centripetal Ad5 movement, arguing against this hypothesis.

A third hypothesis is that kinesins might be needed to counteract the tendency of cytoplasmic dynein alone to accumulate virus particles in the pericentrosomal region, a behavior only slightly increased in LMB-treated cells (Figs 1A and 2B). However, the effect of LMB treatment is much more pronounced upon additional kinesin knockdown, indicating that kinesins do normally contribute to a more dispersed virus distribution. Analysis of Ad5 capsid distribution during the early stages of control cell infection, however, revealed little tendency to congregate at the centrosome prior to reaching the nuclear envelope (Fig 1A), arguing against a requirement for the virus to visit the pericentrosomal region before transport to the nucleus [47].

A final hypothesis raised in this study is that dynein/kinesin-mediated bidirectional Ad5 movement allows capsids to explore the cytoplasm more fully (Fig 6B). Despite a basic role for the motor proteins in linear cargo transport along MTs, we find that they can contribute to less ordered behavior as well, the magnitude of which depends on microtubules as well as Kif5B. We propose, therefore, that a combined role for MT plus end- and minus end-directed MT motor proteins is to permit broad and relatively rapid random-walk exploration of the cytoplasm. We reason that centrosomes, where MT minus ends are concentrated, serve as a cul-de-sac for the virus, which the kinesins normally help to avoid. We observed “exploratory” movements throughout the cytoplasm, consistent with a broader role for a microtubule-based assisted diffusion mechanism in helping virus particles search the entire cytoplasmic space. According to this reasoning, kinesin recruitment should help maintain virus particles in an exploratory state until they interact irreversibly with nuclear pores, thereby facilitating infection. Kif5B knockdown did, in fact, reduce Ad5 infectivity, supporting a role for kinesin-1 in aiding the virus in the early stages of infection.

Another form of exploratory behavior has been observed for vaccinia and baculovirus, which take advantage of the actin polymerization machinery to move within cells, either to reach the nucleus [48] or to spread to neighboring cells [49, 50]. Our data suggest that adenovirus, and perhaps other pathogens may have evolved a distinct microtubule-based mechanism to explore the cytoplasm by recruiting both plus-end and minus-end motors. We also note that diverse forms of vesicular motor protein cargo exhibit relatively random-seeming MT motor-driven movements in nonneuronal cells. Conceivably, this represents another manifestation of cytoplasmic assisted diffusion to enhance the chance of cargoes, such as Golgi vesicles, to encounter sites of docking and fusion with other membranous structures.

Kif5B depletion reduced adenovirus infectivity by only ~20%, as opposed to the effects of nocodazole (>75%) [51] or cytoplasmic dynein light intermediate chain knock down (45%) [19]. We note in this context that the dual effect of kinesin inhibition on Ad motility may minimize the consequences for infectivity. The increased centripetal Ad transport during early infection of Kif5b knockdown cells should increase infectivity, whereas the decrease in assisted diffusion later in infection may inhibit infectivity, with kinesin inhibition suppressing virus exploration of the centrosomal region “in search of” the nucleus. We note that incomplete knockdown of Kif5B and/or the contributions of other kinesin isoforms may also affect the relative magnitude of the two modes of virus transport. Another complicating factor in this analysis is the radial subcellular organization of MTs in the A549 cells used in these studies, and the relatively short distances involved in virus transport to the nuclear surface, especially for viruses that enter the cell at nearby sites within the plasma membrane.

In fact, we expect Kif5B and the other kinesins identified in this study to play a more critical role in differentiated cells (Fig 6C). Polarized lung epithelial cells, similar to other epithelia, have a distinct arrangement of MTs and organelles, with centrosomes located apically and the nucleus basolaterally [52]. MTs tend to have a more columnar apical-basal arrangement [53], as well as a disordered subapical array distinct from the more radial organization in the A549 cells used commonly for adenovirus analysis. We reason that in polarized epithelia cytoplasmic dynein and kinesin-1 may facilitate navigation of Ad along the apico-basal axis of the cell, and, more randomly, through the apical MT meshwork, to allow capsids to find the nucleus efficiently. Further work will be needed to test the role of cytoplasmic dynein and kinesins in adenovirus infection of differentiated cells.

## Materials and methods

### Cells, viruses, chemicals and molecular methods

Human A549 (alveolar basal epithelial; ATCC, CCL-185) and 293A (embryonic kidney; Thermo Fisher, R70507) cells were grown in DMEM supplemented with 10% FBS. Amplification, purification, and labeling of replication-deficient Ad5 were engineered for late GFP expression (courtesy of Dr. Hamish Young, Columbia University, New York, NY) as previously described [5]. Virus infectivity was assessed by fluorescent-focus assays for AdV5-GFP [24]. Antibodies used include mouse monoclonal anti-hexon (Novocastra), anti- γ-tubulin (ab27074 from Abcam), anti-dynein intermediate chain (MAB1618, Millipore), anti-KHC (H1 and H2; MAB1613 and MAB1614, Millipore), rabbit anti-Ad5 (ab6982 from Abcam), anti-GFP (A-11122 from Invitrogen), anti-β-tubulin (ab6046), goat anti-γ-tubulin (sc-7396 from Santa Cruz), anti-Kif5B (sc13356 from Santa Cruz). Mammalian expression constructs used in this work included RFP-tagged Kif5C as well as GFP-tagged full length Kif5A, Kif5B, and KLC1 and KLC2 (all obtained from Kristin Verhey). Plasmids encoding the Ad proteins hexon, 100K and HA-tagged penton base were previously described [5]. Plasmid encoding myc-tagged penton base or protein IX tagged with FLAG and GFP (pIX-flag-GFP) were cloned from purified Ad DNA. Viral DNA was separated from capsid proteins by boiling purified virus particles at 100°C for 5min in 1%SDS and subsequent MiniPrep (Qiagen). Protein IX was cloned into the pEGFP-C1 vector (Clontech, Mountain View, CA) by introducing 5’ EcoRI and 3’ AgeI restriction sites by PCR. The additional C-terminal FLAG-tag was introduced during PCR. Mutations in the sequence and in-frame cloning was tested for by 5’ and 3’ sequencing.

Transient transfections were performed using either Lipofectamine 2000 (Invitrogen) or Effectene (Qiagen). An siGENOME siRNA smartpool custom library targeting 38 plus-end directed human kinesins was designed and purchased from Dharmacon. Other siRNA pools used include Dharmacon siGENOMETM Control Pool and Kif5A, Kif5B, Kif5C, KLC1, KLC2, Kif4A, Kif21A smartpools. siRNA oligonucleotides were transfected into A549 cells using Lipofectamine 2000 (QIAGEN). RNAi-resistant Kif5B was generated using QuickChange II site-directed mutagenesis kit (Agilent technologies).

Adenovirus infections were all performed in a low volume of DMEM containing 2% FBS at 4°C for 30 min to allow virus attachment. The cells were washed three times in cold PBS and incubated in fresh DMEM/2% FBS for 60 min at 37°C, unless stated otherwise, to allow internalization and intracellular transport. Leptomycin B (Sigma) was dissolved in dimethyl sulfoxide (DMSO) and kept at -20°C until use. Cells were treated with 20 nM leptomycin B for 60 min prior to adenovirus attachment and during adenovirus infection.

### Immunofluorescence microscopy and live cell imaging

Cells were grown on glass coverslips and fixed in methanol at -20°C for 5 min. Coverslips were blocked for 30 min in 0.5% donkey serum/PBS; incubated in primary antibody at 37°C for 1 hr, washed, and incubated for 1 hr at 37°C in Cy2-, Cy3-, or Cy5-conjugated secondary antibody; then stained with DAPI for 10 min to visualize DNA. Coverslips were mounted using ProLong Gold antifade mounting medium (Invitrogen) and imaged using either a Leica DM IRB/E inverted microscope equipped with a CCD camera (ORCA 100; Hamamatsu) or an IX80 laser scanning confocal microscope (Olympus FV100 Spectral Confocal System). For all live cell imaging experiments, cells were grown on coverslips in coverslip-bottomed dishes (MatTek Corp.; Ashland, MA) and infected with Alexa-546 labeled adenovirus [5]. Movies were acquired 15–75 min p.i. using a 63X oil immersion objective (actual pixel size ~256 nm/pixel) and a CCD camera (model C9100-12; Hamamatsu) attached to an inverted microscope

(IX80; Olympus; Center Valley, PA), or 60X oil immersion objective (actual pixel size ~109 nm/pixel) and an EMCCD camera (Andor iXon ULTRA 897BV) on a spinning disk confocal microscope (Yokogawa; Olympus). Cells were typically imaged every 2 seconds or 30seconds, as noted. Z-stacks of images covering the entire cell thickness were acquired for the latter analysis, and the total intensity projection is shown. For high temporal resolution particle tracking analysis, imaging was at 23 frames/sec, continuously for 3000 frames. Cell outline and nucleus position in movies was identified from bright field images.

### Ad5 distribution and motility analysis

Cells were judged to exhibit “nuclear accumulation” if they showed > 50% of virus particles associated with nucleus. Cells were judge to exhibit “pericentrosomal accumulation” if virus particles were sufficiently concentrated in this region to colocalize with a centrosomal marker or if centrosome position could be clearly inferred from local adenovirus concentration. To measure the concentration of virus particles in the pericentrosomal area quantitatively, we estimated average adenovirus number within a square of 5.8x5.8 μm^2^ around the centrosome. Relative Number Density (RND), the ratio of virus number density per unit area within the “box” divided by average density throughout the rest of the cytoplasm is calculated as a measure of pericentrosomal virus accumulation.

A custom-tracking algorithm was used to extract the position of adenovirus as a function of time [5]. Cells infected with Alexa-546 labeled viruses were imaged at a temporal resolution of 43ms/frame and Ad5 run length, velocity, and frequencies of movements were determined as previously described [5]. To separate diffusive and active transport events, we fit mean squared displacement (MSD) *vs*. time linearly and quadratically for each track, and used the Schwarz Bayesian information criterion for model selection. Only active transport events, whose MSD increases quadratically with time, were used for run length, velocity, and frequency analysis.

A similar particle tracking program was used to identify Ad5 tracks in movies taken at 2s/frame for 10min (Fig 4F). Most of the tracks in this case had a linearly increasing MSD with time. These tracks, in particular were used to determine apparent diffusion constant (Fig 4J). Values for Ad5 MSD, apparent diffusion constant, and maximum displacement for each track each exhibited an exponential decay distribution, from which the mean and confidence intervals for these parameters were determined.

### Protein and biochemical analysis

Sprague Dawley rat (Taconic) brain tissue was used to prepare rat brain lysate and purified rat cytoplasmic dynein in phosphate-glutamate buffer (pH 7.0) as previously described [25]. Kinesin was enriched for using 5–20% sucrose gradient density centrifugation of the microtubule GTP release fraction. The kinesin-1 fractions were devoid of cytoplasmic dynein and dynactin, and were used for further Ad5 and capsid protein binding analysis. Adenovirus hexon was either recovered from the virus-depleted supernatant from post-lytic cells by immunoprecipitation with a monoclonal anti-hexon antibody [5] or by anion exchange chromatography [54]. Initial steps of penton dodecahedra (Pt-Dd) purification followed the protocol described in [54]. Additionally, the first eluting peak from the anion exchange step was pooled, concentrated using spin columns (Millipore), and applied to a Superose 6 10/300 GL column equilibrated with PBS. Pt-Dd-containing fractions eluted close to the void volume, and were pooled and concentrated. Purified proteins were flash frozen in liquid nitrogen and stored at -80°C.

Mammalian cultured cell lysates were prepared in RIPA buffer (50mM Trizma-maleate, 100 mM NaCl, 1 mM EGTA, [pH 7.4]) containing protease inhibitor cocktail (Sigma) and 1% NP40. Insoluble debris was removed by centrifugation. For immunoprecipitations from cell lysate, antigen was recovered with anti-tag antibodies and protein A sepharose beads (GE Bioscience), washed extensively in RIPA buffer, and analyzed for interactions by immunoblotting. Adenovirus capsid and hexon binding assays were described previously [5]. Briefly, virus or hexon was immunoprecipitated with anti-hexon antibody and protein A sepharose beads from purified virus stock or virus-depleted infected 293A cell lysate, respectively. The beads were washed and incubated for 30 min in Tris-maleate buffer (50 mM Trizma-maleate, 10 mM NaCl, 1 mM EDTA, and 0.1% Tween 20, pH 4.4 or pH 7.4), washed in the same buffer at pH 7.4, and then incubated with purified kinesins at 4°C for 1.5 hr. Following washing, the beads were analyzed for the presence of kinesin by immunoblotting. In reciprocal experiments, anti-kinesin or anti-dynein IC antibodies were used to immunopurify motor proteins, which was subsequently incubated with purified virus capsid stock, Pt-Dd, or 293A cell lysate expressing viral capsid components.

### Statistical analysis

Two-sample comparisons were performed via either Student’s t test unless otherwise specified. F test was used for the comparison of mean run length (Fig 4B), velocity (Fig 4D), apparent diffusion coefficient (Fig 4J), and maximum displacement (Fig 4K), which are assumed to be exponentially distributed. Most error bars shows standard error of the mean, except in Fig 4B, 4C, 4I, 4J and 4K, where a 95% confidence interval is shown. Statistical significance was inferred for P < 0.05 (denoted by *), P<0.01 (denoted by **), or P<0.001 (denoted by ***) for both tests. All statistical tests were performed using MATLAB (MathWorks), Excel (Microsoft) or R software.

## Supporting information

S1 Fig (A) A549 cells were mock transfected or transfected with GFP-Kif5A, GFP-Kif5B or RFP-Kif5C. Cell lysates were blotted with anti-GFP or anti-RFP antibody, and goat-anti-Kif5B antibody. The Kif5B antibody recognized GFP-Kif5B specifically. Relates to Fig 1D. (B) Bee-swarm plot of relative pericentrosomal virus density (see Materials and Methods) from multiple Ad5-infected cells pre-exposed to scrambled and KLC1/2 siRNAs, at 30min p.i., in the presence of LMB. Relates to Fig 2E. (C) Relative pericentrosomal virus density quantification of Ad5 pericentrosomal accumulation in A549 cells treated with leptomycin B (LMB) and siRNAs for kinesin heavy chains implicated in Ad5 transport at 30min p.i. Kif5A, Kif5B, Kif5C, Kif4A, and Kif21A RNAi all lead to increases in pericentrosomal Ad5 accumulation with Kif5B RNAi showing the strongest effect. Relates to Fig 3A. (D) For Kif5B RNAi rescue analysis, A549 cells were treated with Kif5B RNAi and LMB as above, but also transfected with cDNAs encoding RNAi-insensitive kinesin isoforms. Quantification as in (C). Relates to Fig 3B and 3C.
(PDF)

S1 Movie Live recording of labeled adenovirus in LMB treated, control A549 cells between 15 to 75 min p.i. at 30 second intervals. Video relates to Fig 4A.
(MOV)

S2 Movie Live recording of labeled adenovirus in LMB treated, Kif5B knock down A549 cells between 15 to 75 min p.i. at 30 second intervals. Video relates to Fig 4A.
(MOV)

S1 Table Cellular proteins binding directly to the kinesin-1 heavy chain. (DOCX)

S1 References (DOCX)
