# Supplementary figures and images for: Role of kinesins in directed adenovirus transport and cytoplasmic exploration
Source: PLoS Pathog. 2018 May 21;14(5):e1007055. doi: 10.1371/journal.ppat.1007055 (PMC5983873; doi:10.1371/journal.ppat.1007055)

Supplemental Figure 1

(A)

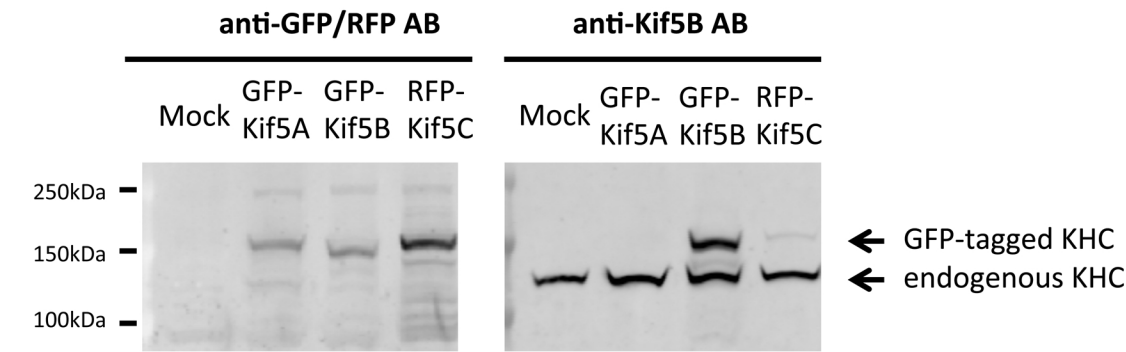

(B)

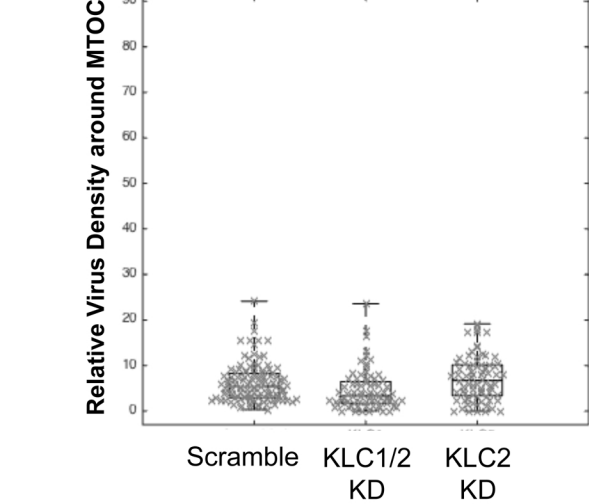

(C)

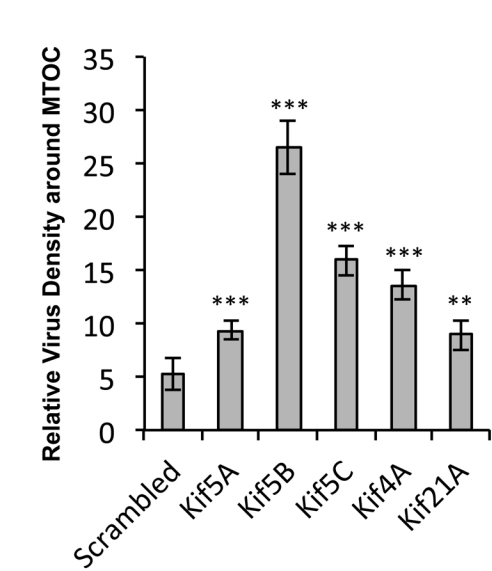

(D)

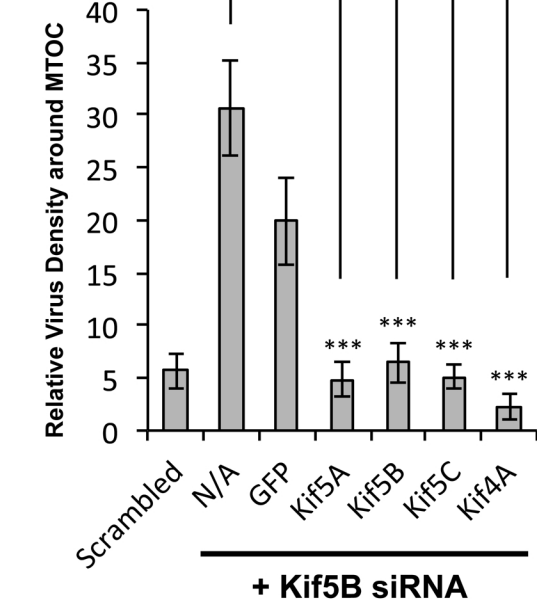

Supplement: S1 Fig — (A) A549 cells were mock transfected or transfected with GFP-Kif5A, GFP-Kif5B or RFP-Kif5C. Cell lysates were blotted with anti-GFP or anti-RFP antibody, and goat-anti-Kif5B antibody. The Kif5B antibody recognized GFP-Kif5B specifically. Relates to Fig 1D. (B) Bee-swarm plot of relative pericentrosomal virus density (see Materials and Methods) from multiple Ad5-infected cells pre-exposed to scrambled and KLC1/2 siRNAs, at 30min p.i., in the presence of LMB. Relates to Fig 2E. (C) Relative pericentrosomal virus density quantification of Ad5 pericentrosomal accumulation in A549 cells treated with leptomycin B (LMB) and siRNAs for kinesin heavy chains implicated in Ad5 transport at 30min p.i. Kif5A, Kif5B, Kif5C, Kif4A, and Kif21A RNAi all lead to increases in pericentrosomal Ad5 accumulation with Kif5B RNAi showing the strongest effect. Relates to Fig 3A. (D) For Kif5B RNAi rescue analysis, A549 cells were treated with Kif5B RNAi and LMB as above, but also transfected with cDNAs encoding RNAi-insensitive kinesin isoforms. Quantification as in (C). Relates to Fig 3B and 3C. (PDF) [file ppat.1007055.s001.pdf]
