# Supplementary material for: Role of kinesins in directed adenovirus transport and cytoplasmic exploration
Source: PLoS Pathog. 2018 May 21;14(5):e1007055. doi: 10.1371/journal.ppat.1007055 (PMC5983873; doi:10.1371/journal.ppat.1007055)
Supplement: S1 Table — (DOCX) [file ppat.1007055.s004.docx]

| protein | binding region | reference |
| --- | --- | --- |
| BicD1 and BicD2 | tail | Grigoriev et al., 2007 |
| casein kinase 2 | motor domain | Schäfer et al., 2009 |
| DISC1 | tail | Taya et al., 2007 |
| dystrobrevin (beta) | tail | Macioce et al., 2003 |
| ensconsin |  | Barlan et al., 2013;  Metzger et al., 2012 |
| Fez1/unc76 | tail | Blasius et al., 2007;  Gindhart et al., 2003 |
| GRIP1 | tail | Setou et al., 2002 |
| HAP1 | tail | Twelvetrees et al., 2010 |
| JIP 1 and JIP 3 | hinge and tail | Fu and Holzbaur, 2013;  Sun et al., 2013 |
| kinectin | tail | Ong et al., 2000 |
| kinesin light chains | tail | Diefenbach et al., 1998 |
| microtubule | tail | Seeger and Rice, 2010 |
| milton | tail | Glater et al., 2006 |
| mNUDC |  | Yamada et al., 2010 |
| mRNP complex | tail | Kanai et al., 2004 |
| myosin Va | tail | Huang et al., 1999 |
| p180 | tail | Diefenbach et al., 2004 |
| penton base (Ad5) | hinge | this study |
| RanPB2 | tail | Cho et al., 2007) |
| SNAP25 | tail | Diefenbach et al., 2002A |
| syntabulin | tail | Cai et al., 2005;  Su et al., 2004 |
| Us11 (herpes simplex virus) | tail | Diefenbach et al., 2002B |
