# Supplementary material for: Role of kinesins in directed adenovirus transport and cytoplasmic exploration
Source: PLoS Pathog. 2018 May 21;14(5):e1007055. doi: 10.1371/journal.ppat.1007055 (PMC5983873; doi:10.1371/journal.ppat.1007055)
Supplement: S1 References — (DOCX) [file ppat.1007055.s005.docx]

**S1 References**

Barlan, K., W. Lu, and V.I. Gelfand. 2013. The microtubule-binding protein ensconsin is an essential cofactor of kinesin-1. *Curr Biol*. 23:317-322.

Blasius, T.L., D. Cai, G.T. Jih, C.P. Toret, and K.J. Verhey. 2007. Two binding partners cooperate to activate the molecular motor Kinesin-1. *J Cell Biol*. 176:11-17.

Cai, Q., C. Gerwin, and Z.H. Sheng. 2005. Syntabulin-mediated anterograde transport of mitochondria along neuronal processes. *J Cell Biol*. 170:959-969.

Cho, K.I., Y. Cai, H. Yi, A. Yeh, A. Aslanukov, and P.A. Ferreira. 2007. Association of the kinesin-binding domain of RanBP2 to KIF5B and KIF5C determines mitochondria localization and function. *Traffic*. 8:1722-1735.

Diefenbach R.J., J.P. Mackay, P.J. Armati, and A.L. Cunningham. 1998. The C-terminal region of the stalk domain of ubiquitous human kinesin heavy chain contains the binding site for kinesin light chain. *Biochemistry*. 37:16663-70.

Diefenbach, R.J., E. Diefenbach, M.W. Douglas, and A.L. Cunningham. 2002. The heavy chain of conventional kinesin interacts with the SNARE proteins SNAP25 and SNAP23. *Biochemistry*. 41:14906-14915.

Diefenbach, R.J., M. Miranda-Saksena, E. Diefenbach, D.J. Holland, R.A. Boadle, P.J. Armati, and A.L. Cunningham. 2002. Herpes simplex virus tegument protein US11 interacts with conventional kinesin heavy chain. *J Virol*. 76:3282-91.

Diefenbach, R.J., E. Diefenbach, M.W. Douglas, and A.L. Cunningham. 2004. The ribosome receptor, p180, interacts with kinesin heavy chain, KIF5B. *Biochem Biophys Res Commun*. 2;319(3):987-92.

Fu, M.M., and E.L. Holzbaur. 2013. JIP1 regulates the directionality of APP axonal transport by coordinating kinesin and dynein motors. *J Cell Biol*. 202:495-508.

Gindhart, J.G., J. Chen, M. Faulkner, R. Gandhi, K. Doerner, T. Wisniewski, and A. Nandlestadt. 2003. The kinesin-associated protein UNC-76 is required for axonal transport in the Drosophila nervous system. *Mol Biol Cell*. 14:3356-3365.

Glater, E.E., L.J. Megeath, R.S. Stowers, and T.L. Schwarz. 2006. Axonal transport of mitochondria requires milton to recruit kinesin heavy chain and is light chain independent. *J Cell Biol*. 173:545-557.

Grigoriev, I., D. Splinter, N. Keijzer, P.S. Wulf, J. Demmers, T. Ohtsuka, M. Modesti, I.V. Maly, F. Grosveld, C.C. Hoogenraad, and A. Akhmanova. 2007. Rab6 regulates transport and targeting of exocytotic carriers. *Dev Cell*. 13:305-314.

Huang, J.D., S.T. Brady, B.W. Richards, D. Stenolen, J.H. Resau, N.G. Copeland, and N.A. Jenkins. 1999. Direct interaction of microtubule- and actin-based transport motors. *Nature*. 397(6716):267-70.

Kanai, Y., N. Dohmae, and N. Hirokawa. 2004. Kinesin transports RNA: isolation and characterization of an RNA-transporting granule. *Neuron*. 43:513-525.

Macioce, P., G. Gambara, M. Bernassola, L. Gaddini, P. Torreri, G. Macchia, C. Ramoni, M. Ceccarini, and T.C. Petrucci. 2003. Beta-dystrobrevin interacts directly with kinesin heavy chain in brain. *J Cell Sci*. 116(Pt 23):4847-56.

Metzger, T., V. Gache, M. Xu, B. Cadot, E.S. Folker, B.E. Richardson, E.R. Gomes, and M.K. Baylies. 2012. MAP and kinesin-dependent nuclear positioning is required for skeletal muscle function. *Nature*. 484:120-124.

Ong, L.L., A.P. Lim, C.P. Er, S.A. Kuznetsov, and H. Yu. 2000. Kinectin-kinesin binding domains and their effects on organelle motility. *J Biol Chem*. 275(42):32854-60.

Schäfer, B., C. Götz, J. Dudek, A. Hessenauer, U. Matti, and M. Montenarh. 2009. KIF5C: a new binding partner for protein kinase CK2 with a preference for the CK2alpha' subunit. *Cell Mol Life Sci*. 66(2):339-49.

Seeger, M.A., and S.E. Rice. 2010. Microtubule-associated protein-like binding of the kinesin-1 tail to microtubules. 2010. *J Biol Chem*. 285(11):8155-62.

Setou, M., D.H. Seog, Y. Tanaka, Y. Kanai, Y. Takei, M. Kawagishi, and N. Hirokawa. 2002. Glutamate-receptor-interacting protein GRIP1 directly steers kinesin to dendrites. *Nature*. 417:83-87.

Su, Q., Q. Cai, C. Gerwin, C.L. Smith, and Z.H. Sheng. 2004. Syntabulin is a microtubule-associated protein implicated in syntaxin transport in neurons. *Nat Cell Biol*. 6:941-953.

Sun, E., J. He, and X. Zhuang. 2013. Live cell imaging of viral entry. *Curr Opin Virol*. 3:34-43.

Taya, S., T. Shinoda, D. Tsuboi, J. Asaki, K. Nagai, T. Hikita, S. Kuroda, K. Kuroda, M. Shimizu, S. Hirotsune, A. Iwamatsu, and K. Kaibuchi. 2007. DISC1 regulates the transport of the NUDEL/LIS1/14-3-3epsilon complex through kinesin-1. *J Neurosci*. 27:15-26.

Twelvetrees, A.E., E.Y. Yuen, I.L. Arancibia-Carcamo, A.F. MacAskill, P. Rostaing, M.J. Lumb, S. Humbert, A. Triller, F. Saudou, Z. Yan, and J.T. Kittler. 2010. Delivery of GABAARs to synapses is mediated by HAP1-KIF5 and disrupted by mutant huntingtin. *Neuron*. 65(1):53-65.

Yamada, M., S. Toba, T. Takitoh, Y. Yoshida, D. Mori, T. Nakamura, A.H. Iwane, T. Yanagida, H. Imai, L.Y. Yu-Lee, T. Schroer, A. Wynshaw-Boris, and S. Hirotsune. 2010. mNUDC is required for plus-end-directed transport of cytoplasmic dynein and dynactins by kinesin-1. *EMBO J*. 29:517-531.
